# Abundance of Sea Kraits Correlates with Precipitation

**DOI:** 10.1371/journal.pone.0028556

**Published:** 2011-12-14

**Authors:** Harvey B. Lillywhite, Ming-Chung Tu

**Affiliations:** 1 Department of Biology, University of Florida, Gainesville, Florida, United States of America; 2 Department of Life Science, National Taiwan Normal University, Taipei, Taiwan; University of Sao Paulo, Brazil

## Abstract

Recent studies have shown that sea kraits (*Laticauda* spp.) – amphibious sea snakes – dehydrate without a source of fresh water, drink only fresh water or very dilute brackish water, and have a spatial distribution of abundance that correlates with freshwater sites in Taiwan. The spatial distribution correlates with sites where there is a source of fresh water in addition to local precipitation. Here we report six years of longitudinal data on the abundance of sea kraits related to precipitation at sites where these snakes are normally abundant in the coastal waters of Lanyu (Orchid Island), Taiwan. The number of observed sea kraits varies from year-to-year and correlates positively with previous 6-mo cumulative rainfall, which serves as an inverse index of drought. Grouped data for snake counts indicate that mean abundance in wet years is nearly 3-fold greater than in dry years, and this difference is significant. These data corroborate previous findings and suggest that freshwater dependence influences the abundance or activity of sea kraits on both spatial and temporal scales. The increasing evidence for freshwater dependence in these and other marine species have important implications for the possible impact of climate change on sea snake distributions.

## Introduction

Water is essential to organisms, which have evolved numerous adaptations for maintaining adequate body water content. Two especially challenging environments are terrestrial deserts and marine habitats in which freely available fresh water is in short supply or absent. It was long thought that sea snakes and other marine reptiles obtained water directly or indirectly from sea water, using extrarenal salt glands to eliminate excess salt. It is now clear, however, that amphibious sea kraits (*Laticauda* spp.) dehydrate without a source of fresh water and require fresh water for water balance [1], [2]. Moreover, studies of sea kraits at Lanyu (Orchid Island), Taiwan, demonstrate that the spatial distribution of abundance correlates with sites where there is a source of fresh water other than direct rainfall, or where fresh water is abundant [2].

Here we report six years of longitudinal data on the abundance of sea kraits related to precipitation at Lanyu, Taiwan. In 2007 we observed that counts of sea kraits were consistently lower than in previous years, and these coincided with unusual drought on the island. Some of the villages ran out of water, and many of the streams or springs that typically discharged into the ocean at coastal sites were dry. Thus, we continued to count snake abundance in subsequent years, and we compared previous years' data with precipitation data as an index of drought. We tested the hypothesis that sea krait abundance correlates positively with cumulative rainfall, which serves as an inverse index of drought.

## Methods

### Ethics Statement

These investigations were carried out within guidelines and approval of the respective institutional animal care and use committees of National Taiwan Normal University and the University of Florida (IACUC approvals E528 and 200902798). Observations of sea kraits were also approved by the Orchid Island administrative office.

### Study Sites and Snake Counts

During the six years of this study we counted snakes at three sites where sea kraits were relatively most abundant [2]. Counts of sea kraits included three species that are common at Lanyu: *Laticauda colubrina*, *L. laticaudata*, and *L. semifasciata*. We counted snakes during the months of June and July when snakes are similarly active. At each site, the abundance of sea kraits was estimated by the collective sightings of 2–5 persons who searched tidal pools, adjacent rocks, and shallow coastal areas for one hour during evenings after dark when the snakes were most active. During repeated visits to various sites, we consistently covered the same tidal pools, inlets and rocks. We previously found there was no correlation between the number of persons searching and the number of snakes observed [2], demonstrating that increasing the number of people did not increase the number of snakes that were found.

**Figure 1:**
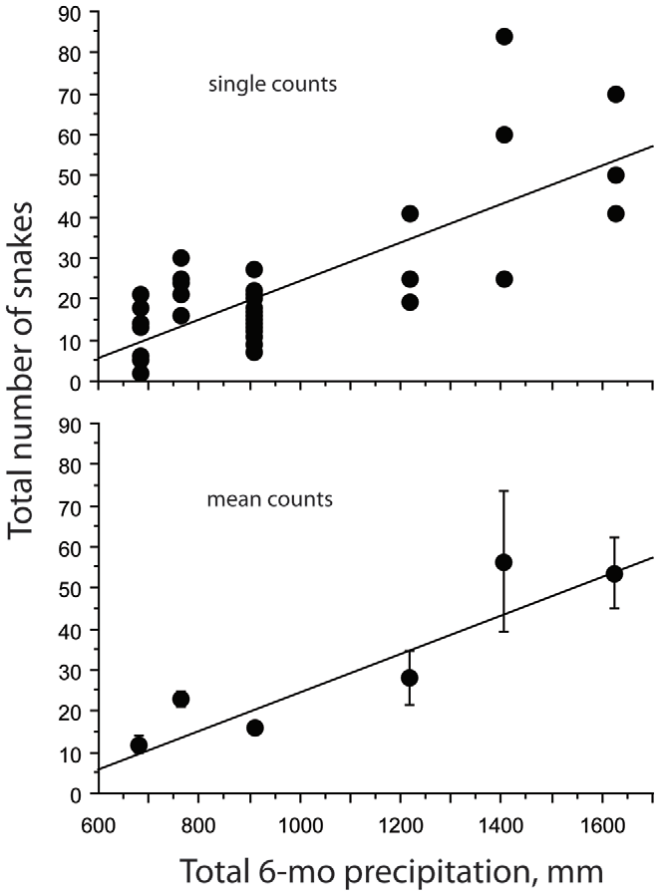
Relationship between total numbers of sea kraits counted on different nights during June/July and the cumulative precipitation during the previous six months. The upper graph illustrates data for single counts, and the lower graph plots mean ± SE (R^2^ = 0.575, P<0.0001).

For each year with snake count data we totaled the cumulative precipitation that occurred during the previous six months, which represents an index for comparing relative “wet” or “dry” years [2]. We then used standard regression analysis (StatView 5.0.1) to examine the relationship between the abundance of snakes and the previous 6-month total precipitation. The abundance of snakes was represented by combining the total number seen for all species at a given count, and plotting the data for all counts at all sites (Fig. 1). Precipitation data were obtained from records kept at the weather station on Lanyu.

Because temperature also determines sea snake distributions [3], [4], we computed mean coastal water temperatures at Orchid Island from surface water temperatures recorded from 38 sites during June and July of each year. Again we used standard regression analysis to examine the relationship between the mean abundance of snakes and the mean water temperatures recorded (June + July) for the years of the study.

## Results

Nightly counts of snakes varied from as few as 5 to as many as 84, totaled for all three species. Of the three species, *Laticauda semifasciata* was the most abundant. For example, during counts made at two sites in 2010, *L. semifasciata* accounted for 93.7% and 89% of all snakes that were seen. This pattern was generally similar in other years, but the species identities were not always known with certainty. Therefore, our data represent all three sea krait species that were present at the coastal sites during our counts. A mating ball involving 37 *L. semifasciata* was observed on one occasion during 2010, but we did not include this number in the data that we analyze here.

Precipitation was totaled for six months prior to the earliest month when we counted snakes. These totals varied from 684 mm during the driest year (2007) to 1627 mm during the wettest year (2006). We did not monitor the salinity of ocean water at the coastal sites, but we noted that streams and a spring feeding into the ocean at the sites were completely dry during June and July of 2007.

**Figure 2:**
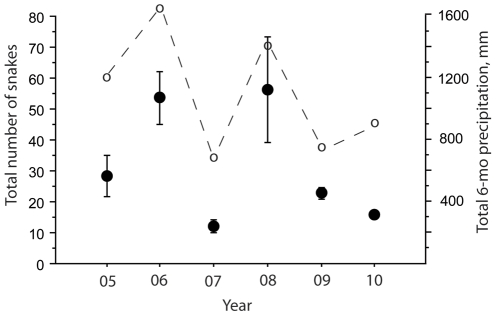
Year-to-year variation in the mean number of snakes counted (± SE) and previous 6-mo precipitation during the six years of the study.

**Figure 3:**
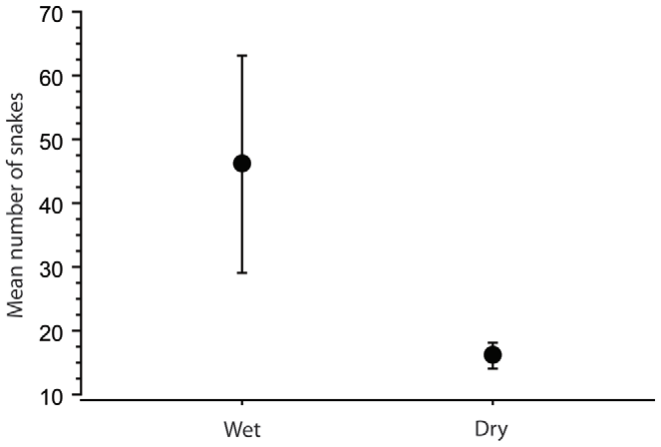
Mean number of snakes counted during wet (>1000 mm, 6 mo precipitation, N = 3) and dry (<1000 mm, 6 mo precipitation, N = 3) years. Vertical bars denote 95% confidence intervals for the mean of counts.

Regression analysis demonstrates there is a positive relationship between the mean of total snakes counted and the cumulative 6-mo prior precipitation in different years (F = 60.79, P<0.0001) (Fig. 1). Thus, the relative abundance of sea kraits varies from year–to–year, depending on the amount of rainfall (Fig. 2). Based on Fig. 2, we divided the snake counts between wet years (>1000 mm) and dry years (<1000 mm). The mean number of sea kraits counted in wet years was significantly greater than the mean count for dry years (Mann Whitney U test, P<0.0001) (Fig. 3).

Mean ocean temperatures (°C) in the coastal waters of Lanyu varied from 29.23±0.07 SE in 2009 to 29.84±0.10 in 2007, and all year–to–year variation was <1°C. The very small annual differences in ocean temperature were not correlated with differences in sea krait abundance (F = 0.010, R^2^ = 0.003, P = 0.9233).

## Discussion

Here we demonstrate that the year-to-year abundance of sea kraits varies in relation to precipitation. We qualify this statement to make clear that ‘abundance’ refers to the counts of visually detected snakes that are active at night in shallow coastal waters or on rocks in the intertidal zone. Sea kraits at Lanyu are most active in these locations at night, and our counts involved prescribed nocturnal searches at locations where snakes were known to be abundant.

Correlations between abundance or activity of snakes and precipitation are well known in terrestrial species [5]–[8]. The changes in conspicuous activity of snakes related to rainfall are likely to be causal in three contexts. First, when terrestrial conditions become dry snakes may remain secluded in below-ground refugia where losses of body water are mitigated, whereas above-ground activity would result in greater deficits of water balance. Second, wetter conditions related to rainfall may affect the prey of snakes (especially frogs), so their increased activity might be related to foraging, prey capture, and growth. Third, wetter conditions will tend to lower the ambient temperatures, and lower temperatures might be important for snakes that are active in seasonally hot environments such as deserts.

The relation between precipitation and abundance or activity is poorly documented in sea snakes and requires further explanation. Previously we demonstrated that sea kraits drink fresh water and are dependent on fresh water to remain in water balance [2]. Freshwater drinking by sea kraits also is well known in behavioral and ecological contexts, and dehydrated individuals have been found in both terrestrial and marine settings [1], [2], [9], [10]. At New Caledonia, sea kraits emerge from terrestrial refugia and crawl on top of rocks or other locations to drink rain water that collects in small pools when there is significant rain following a period of drought [1]. In the laboratory, sea kraits dehydrate in sea water, and they drink fresh water or very dilute brackish water to rehydrate [2]. They do not drink seawater, and evidently the salt glands of these snakes do not have the capacity to secrete sufficient salt in order to make a water profit directly from sea water [2].

What happens to sea kraits when the observed numbers are low? It seems unlikely that declines in abundance are attributable to mortality, for the snake numbers rebound in successive years (Fig. 2). Note, for example, that the number of snakes observed during 2008 is roughly the same (but somewhat higher) than what was observed in 2006, the number decreasing to about 20% of these values in the drier intervening year of 2007 (Fig. 2). Nearly all of the snakes that we counted were adults, so year-to-year increases in number could not possibly represent recruitment of young related to a ‘bubble’ in reproduction. Insofar as sea kraits are susceptible to dehydration, mortality could be an unknown part of the declines during dry years, with immigration making up the difference in wet years. It is unlikely that snakes migrate long distances to sources of fresh water, which might be unreasonable distances anyway. Moreover, sea kraits have been shown to have strong site fidelity, and translocated individuals have strong tendencies to return to specific locations and specific stretches of beach [11].

We propose that the likely survival of sea kraits during drier periods is attributable to some combination of two factors. One is a localized relocation over shorter distances to possible freshwater sources, and the other is a reduction in activity related to seclusion in moister terrestrial refugia or lesser exposure in the terrestrial zone altogether. Both of these behaviors might reduce visual sightings of snakes at the intertidal. As an example of one possibility, numerous sea kraits take refuge and also reproduce inside a sea cave that has a narrow opening at intertidal level [12]. Such sea caves can generate fresh water from the compression and rarefaction of the air associated with the oceanic swell that rolls into the mouth of a cave. The mist forms and disappears periodically in synchrony with the waves. The mist condenses on the walls and fills the shallow pools, which contribute water-saturated air to the chamber in the cave. The coastline of Lanyu is rocky with cavernous spaces that might provide numerous such caves or pockets wherein sea kraits might seclude themselves. During dry conditions these snakes conceivably respond to heavy rainfall by detecting changes of pressure related to barometric pressure fluctuations [12]. This, in turn, would signal an eventual return to more usual and dispersed favorable sites, such as those that we sampled.

Sea kraits at Orchid Island do not appear to move very far inland to occupy other terrestrial refugia outside the intertidal zone, but this is a possibility. In New Caledonia, sea kraits (*Laticauda saintgeronsi*) seek refuge beneath rocks or in burrows during dry weather [1], and we have observed *L. guineai* and *L. colubrina* in similar refugia some tens of meters from shoreline on smaller islands of Papua New Guinea and Fiji. The timing of activity such as crossing beaches to engage foraging trips to sea occur largely during hours of dark, but sea kraits exhibit more conspicuous behaviors during rainfall evidently due to the importance of finding fresh water that drips from vegetation and forms temporary pools on rocks [1]. Otherwise, sea kraits can become severely dehydrated during periods of drought [2].

It is now clear that sea kraits have reliance on fresh water, and this aspect of physiology can therefore determine (i) local abundance in space, (ii) changes in abundance through time, and (iii) possible long-term changes in abundance – including extinctions – if climate change produces weather anomalies that reduce precipitation [13], [14]. It seems inevitable that freshwater dependence will be shown to influence broader patterns of distribution for these and possibly other taxa of marine vertebrates.
